# A Methodological Framework for Developing More Just Footprints: The Contribution of Footprints to Environmental Policies and Justice

**DOI:** 10.1007/s11948-019-00100-8

**Published:** 2019-03-28

**Authors:** Rita Vasconcellos Oliveira

**Affiliations:** Programme for Applied Ethics, Department of Philosophy and Religious Studies, Norwegian University of Science and Technology (NTNU), Dragvoll, 7491 Trondheim, Norway

**Keywords:** Footprint, Environmental policy, Environmental justice, Climate change, Land use, Water use

## Abstract

The rapid growth of human population and associated industrialisation creates strains on resources and climate. One way to understand the impact of human activity is to quantify the total environmental pressures by measuring the ‘footprint’. Footprints account for the total direct and/or indirect effects of a product or a consumption activity, which may be related to e.g. carbon, water or land use, and can be seen as a proxy for environmental responsibility. Footprints shape climate and resource debates, especially concerning environmental strategies. However, in general, footprints hold a dichotomous producer–consumer perspective that is not unanimously accepted. In addition, the current footprinting system transmits a simplistic message about environmental responsibility that taints the justice debate and jeopardises the validity of policies based on them. Consequently, it is crucial to question who is (and should be) accountable for adverse environmental effects. It is also critical to investigate how the methodological characteristics of footprints shape and affect the efficacy of policies on climate and natural resources. This article examines these challenges, focusing on negative justice and policy implications resulting from assigning environmental responsibility to a sole agent. The article proposes, and morally justifies, the development of a footprinting method that includes justice parameters in an attempt to render fair results that are more meaningful for environmental action. The second objective is to establish the potential of this new framework to promote environmental responsibility and justice while facilitating policymaking. The suggested justice elements aim at turning footprints into a concrete environmental policy instrument framed under the value of environmental fairness.

## Introduction

Climate change coupled with other challenges such as the natural resource crisis creates the need for detailed information about present and future environmental scenarios. The way such scenarios are created influences their results, which in turn, shape policies that affect populations and groups differently. Thus, environmental scenarios structure policies that can create or sustain asymmetries either in terms of access to resources or in the management of climate change impacts. The scientific and ethical communities are called to the task of advancing not only scientifically solid methods of understanding the state of environmental affairs but also of creating fair assessment tools. Among other requisites, environmental assessment methods should make clear who is held responsible for environmental stresses, as well as to what degree (Finnveden and Moberg 2005). Only by attributing environmental impacts in this way can sustainable policies be put in place, and environmental justice promoted.

Environmental indicators are a key tool used in environmental assessment methods. These indicators were developed in direct response to the challenge of comprehending and quantifying human impact on Earth. Generally, an environmental indicator is ‘a parameter or a value derived from parameters that points to, provides information about and/or describes the state of the environment’ (OECD 2001). The advantage of using an indicator is the possibility of translating the state of a very complex system into humanly digestible information.

Footprints, such as resource and climate footprints, are important examples of descriptive environmental indicators since they attempt to characterise environmental states or changes of a particular environmental component. These footprints aim at e.g. accounting for GHG (greenhouse gas) emissions (via the carbon footprint), water use (via the water footprint) and the impact on land (via the land-use footprint). The footprints considered are resource and climate footprints, calculated using data organized in MRIO (multi-region input–output) tables. Footprints are commonly applied to describe the impact of humans on ecosystems, i.e. they attribute environmental impacts, emissions or resource consumption to economic activity. Traditionally, the environmental justice debate around resource use is associated with differential access to and quality of resources according to geographical (Cutter et al. 1996), gender (Ahlers and Zwarteveen 2009), social (Jenerette et al. 2011) or generational boundaries (Martinsen and Seibt 2013; Vasconcellos Oliveira 2018). In order to address such topics, it is invaluable to know the state of affairs of resource use and distribution. Furthermore, correct information about resource scarcity helps in developing concrete strategies to reduce inequality.

Footprints integrate the scientific discourse that stretches from subjects of economy (Ferng 2003) and life sciences (Wilting et al. 2017) to engineering (Lawlor and Morley 2017). Since footprints are commonly used to support and promote particular scientific, engineering and behavioural options, these particular indicators are relevant tools for scientific dissemination (Lee 2015; Milford et al. 2013). Additionally, footprints are communication instruments to a wider audience (Hammond 2007) with a concrete influence on public opinion (Care2 2018). For example, both scientific networks (Global Footprint Network 2018a, b) and NGOs use footprints as tools for increasing environmental awareness in citizens, companies and economic sectors (WWF 2018; Greenpeace International 2017).

Footprints are neither morally neutral indicators nor used impartially in environmental discourse (Martinez-Alier et al. 2014; Nerlich and Koteyko 2009). Nonetheless, the development and use of footprints are highly politicised, influencing the sustainability dialectic about resources and environmental impacts (European Comission 2008; EPA 2017). Footprints are thus both scientific instruments and policymaking tools, and integrate the justification for environmental policies as an important interface between scientists and politicians (European Comission 2016). Information derived from footprint accounting, both directly and indirectly, influences policies which affect nations and communities differently, laying the ground for environmental (in)justice. This is the case for environmental impact accounting and potential taxation in the European Union (EU). As Ekins et al. (2011) note, a considerable number of European households would need additional support to overcome the negative economic impacts of an environmental tax reform. Similar conclusions hold for carbon taxation on disadvantaged population groups (Dennig et al. 2015). Another example of the prominence of footprints in political discourse is the EU’s Sustainable Consumption and Production Action Plan (European Comission 2008). The EU Commission established this methodology for product and sector environmental assessment and looks for further interconnection of footprints with environmental EU strategies. Furthermore, the European Commission supports the use of footprints as instruments of communication on environmental performance (European Comission 2016). In the North American context, footprints are not so relevant politically; nevertheless, the US Environmental Protection Agency (EPA) supports sustainable development initiatives that are based on their information (EPA 2017). On a global level, there is the example of several indicators for the United Nations (UN) sustainable development goals which are footprints (e.g. indicator 8.4.1. material footprint) (UN 2016). All in all, the application of footprints both in policy and science underscores ethical tensions that should be addressed not only by the agents who generate information but also by those who use it: i.e. scientists, engineers and politicians.

One relevant ethical ramification of utilising footprints is the consideration of environmental responsibility. In the environmental justice tradition, environmental responsibility is attributed to a wide variety of agents (Schlosberg 2009; Monsma 2006; Middlemiss 2010). However, with the extensive and continuous application of footprints to climate and resource debates (Hayward 2006; Kolers 2012; Terry 2009), particular agents are often singled out as directly accountable for the source use and/or impacts, diverting attention from other possible actors. This creates additional environmental responsibility asymmetries that then affect nations and societal groups differently. For this reason, it can be argued that scientists and engineers are morally responsible for the implications of the environmental impact tools they use in their research.

So far, environmental and justice indexes developers have been more sensitive than indicator creators to the argument of designing environmental quantitative measures that include environmental justice elements. Sets of indicators and indexes have been elaborated that focus on specific targets, such as sustainable energy (Davidsdottir et al. 2007), environmental quality (e.g. Environmental Performance Index) and human wellbeing (Prescott-Allen 2001). In other cases, indexes were created to quantify more general concepts such as sustainable development (e.g. Eurostat Sustainable Development Index). Nevertheless, as Sarah Fredericks (2013: 351) notes, there is not a single index that includes all significant environmental, social and economic elements and allows monitoring of the distribution of environmental benefits or burdens within a nation or community. Thus, at the current state of development, indexes cannot portray a complete picture of environmental justice in the landscape of nations and communities. The same is true with sets of indicators. Nevertheless, when applied in particular contexts, sets of indicators provide relevant data that help to successfully describe and assess specific justice dimensions such as vulnerability to environmental factors (e.g. relating to climate or pollution).

Nevertheless, despite providing more specific (and limited) information when compared with indexes or sets of indicators, sole environmental footprint indicators still have great policy potential. They can contribute to a more accurate and nuanced picture of the present and future distributive situations. However, if calculated without justice concerns, they contribute to the perpetuation of distributive and environmental unfairness (Fredericks 2013). Footprints are therefore well-suited for assessing the (national and regional) provenance of present (and future) emissions, impacts or resource uses, and for pinpointing the agents responsible for those effects. A more just distribution of the encumbrances of environmental change and the setting of balanced emissions, impacts and resource uses depends directly on the results from environmental indicators. It is reasonable to state that societal action and policymaking based on accurate information helps (re)establish the grounds for distributive justice. Distributive justice here concerns the division of benefits and burdens among citizens. The justifications for such distribution are based on moral arguments which serve to guide political processes and structures (Roemer 1998). Environmental, climate and resource justice are only possible if policies and societal action adequately address scientific evidence. Conversely, for scientific conclusions to be properly integrated into the environmental discourse, it is vital to understand the ethical implications of the methods currently used in sustainability assessment, or there is a risk for misinterpretation.

In this article, one of the key arguments is that environmental assessment—in this case, via the footprint method—shapes environmental policies and societal actions. This is considered under the polarised debate on climate and resource use within the context of environmental justice (Schlosberg and Collins 2014). More just methods of assessing the state of resources and of the environment have the potential to steer policies towards increased environmental justice, which is needed in the areas of climate and resource use (Figueroa and Mills 2001, Schlosberg and Collins 2014). Socio-political actions, such as a resource tax, have the potential to favour either the disenfranchised or sustain current environmental and social inequalities (Crisp and Jamieson 2000). Both the developers of footprint accounting methods (e.g. natural scientists, economists) and the users (e.g. engineers, politicians) are morally responsible for the outcomes of actions based on the numbers generated by footprints. Consequently, they are morally obliged to develop and apply methods of environmental assessment that provide a more correct picture of reality and give the correct extent of responsibility to the correct agents. As Fredericks (2013: 6) writes, some indexes theorists have recognized that normative priorities play a role in index development and a few authors even recognize the need to consider different ethical perspectives in this development. The same reasoning can be applied to indicators and to those who work with them and on their development.

This article goes beyond the examination and exploration of the justice and policymaking implications of footprints, and establishes ethical principles for the development of a renewed framework. Contrary to the ‘multiple accounting’ that Steininger et al. (2016) suggest (for carbon accounting), proposed here is a single novel theoretical framework for assigning environmental responsibility framed by justice concerns. The aim is to establish a procedural framework for footprint calculation based on justice grounds. Here, the article assumes a broad interpretation of environmental justice which integrates the classical distributive (Shrader-Frechette 2002) and participatory issues (Figueroa and Mills 2001), and also includes developmental and global facets (Schlosberg 2009). In the case of developmental justice, it includes (climate and resource) conditions and models for the fair socio-economic development of nations and individuals (Ray 1998) and in the case of global justice it includes the national and the supra-national (climate and resource) justice dimensions, and their relations and interactions (Pogge 2001). The ultimate objective of this article is to make footprints a morally sound (just) tool for environmental responsibilisation of agents, and to strengthen the influence of scientific information in developmental, global and distributive justice contexts.

## The Justice Repercussions of Using Footprints

Footprint calculations can be divided into two types: production-based and consumption-based. Each supports both scientific (Weinzettel et al. 2013; Steen-Olsen et al. 2012) and policy discourses on sustainability (UNESCO 2009; UN PBSO 2012), with a direct impact on environmental justice debates, especially in relation to global justice and distributive inequalities (Hayward 2006). However, within these categories, footprint calculation can differ a great deal (Hoekstra et al. 2011; Wiedmann and Minx 2008) which means that results and conclusions can vary significantly. These differences can lead to contrary discourses about who (individually and collectively) should change and support actions to mitigate and prevent further environmental degradation. Striking illustrations of how footprints have controversial results and applications are, for example, the discussion around the (non-) inclusion of rain-fed agriculture in water footprint accounting (Aldaya et al. 2010) or the inconsistency of metrics of the same footprint (Hoekstra 2016). Land and carbon footprints are also not immune to contentious disputes affecting the acceptability of their results in wider environmental impact debates, for example concerning the emissions of toxic substances that are not related to climate change impacts, or lack of applicability in governance issues (Laurent et al. 2012; Kaphengst 2014).

The aim in what follows is to make clear which agent is held responsible for environmental effects when employing footprints and the ramifications for environmental justice of footprint accounting. The determination and evaluation of an agent’s responsibility for their actions (justice agency) and the potential for mitigating or eliminating environmental impacts depend directly on a common understanding of what footprints actually determine. The ethical implications of adopting current calculation methods for developmental and global justice are also addressed, in an attempt to provide a rationale for the development of a different footprint methodology. For expediency, only carbon is used as an example since land and water footprints can also be calculated by both accounting methods.

## The Responsibility Duality: Producers as the ‘Scapegoats’

Production-based accounting sets system boundaries within a geographically or organisationally defined area, meaning that only the use, emissions and/or impacts coming from activities within those frontiers are included. Production-based footprints thus account for uses, impacts and/or emissions that occur directly during production or operation of goods or services but not in the supply chain. This accounting method allocates the environmental (resource use, emissions or impacts) responsibility solely to the agent that originates energy, goods or services, i.e. exclusively to the producer. The approach is favoured by several international institutions such as the Intergovernmental Panel on Climate Change (IPCC) or the World Resources Institute (Garg et al. 2006; WRI and WBCSD 2004). The Kyoto Protocol also sanctioned this approach (for CO_2_). Every nation reports their GHG emissions to the United Nations Framework Convention on Climate Change (UNFCCC) under the production-based accounting approach (Garg et al. 2006; UNFCCC 2004); the emissions are consequently the basis for international global (carbon) targets.

It is argued here that the application of the production-based principle has created a significant political effect in the way nations are perceived, particularly because it is now clear that some countries may hardly (or ever) be able to achieve the international established carbon targets (Munksgaard and Pedersen 2001). This situation creates the risk of decreased international support to such nations in the case of environmental disasters, especially related to climate change, and leads to stigmatisation of developing countries with an economy based on carbon (and/or resource) intensive manufacture. If there is ‘evidence’ (e.g. national carbon footprints) that these countries are main contributors to the phenomenon of global warming (Hertwich and Peters 2009), the chances of international solidarity can dramatically diminish. Moreover, international aid agencies have a far more difficult task justifying support to these victims when there are many other countries in distress that apparently have not “caused” their own misfortune. Furthermore, as demonstrated in the literature, the societal groups most affected by environmental catastrophes are also the ones suffering most from socioeconomic inequalities (Field et al. 2012). Countries such as China have increased the general level of their population’s wellbeing mainly through the creation of jobs in or related to industries with high environmental impact (Elliott and Shanshan 2008). This job creation was concentrated in some nations and regions, while some population groups economically deprived gained new sources of income due to such industries. Consequently, the wellbeing of these groups is highly dependent on industries that are major sources of environmental impact.

The method also leads to the rapid change in environmental impact profiles of nations in the last years, mainly associated with carbon. Scientists suggest that the course of international policy on climate, triggered by production-based carbon accounting, induces ‘carbon leakage’ (Eichner and Pethig 2011; Reinaud 2008), i.e. the phenomenon where businesses, due to increased costs related to climate policies, transfer production to countries with laxer constraints on GHG emissions. As Reyer Gerlagh and Onno Kuik (2014: 386–387) show, in an optimistic scenario, “the rate of carbon leakage is 9.5%; 40% of the relocated CO_2_ emissions leak to developing countries, 34% to OECD countries, and 26% to countries of the former Soviet Union.” The EU has concerns about this phenomenon since it can potentially lead to an increase in global emissions, and is a problem in key energy-intensive industries (European Comission 2018).

Evidence shows that during the last decades, many polluting and/or resource intensive industries indeed moved from richer countries to developing nations, reinforcing the idea that stricter environmental policy causes the delocalisation of such industries (Jänicke et al. 1997). There is a serious shift in the national emissions profile of nations that are committed to the Kyoto protocol as demonstrated by Barker et al. (2007) for the EU primary aluminium sector, and by Aichele and Felbermayr (2015) through bilateral trade. Kyoto protocol abiding countries have increased the importation of goods and services that were produced with high carbon emissions. These imports come from non-committed countries. By trading this way, Kyoto protocol abiding countries increased the emission intensity of their imports (Aichele and Felbermayr 2015). Meanwhile, other footprints of developed countries (e.g. EU) have also decreased and allowed some of them to reach their targets. Nevertheless, the real reason behind their ‘success’ may sometimes be defined as ‘pollution’ leakage (Paltsev 2001).

Assuming ‘carbon leakage’ and ‘pollution leakage’ to be true, there are relevant justice implications in addition to the environmental ones (Smarzynska and Wei 2001). Delocalization of heavy carbon emitters generates negative (local and regional) social (Pickles and Smith 2010) and economic effects (Dunford et al. 2013), in developing and developed countries. This situation deepens international developmental asymmetry and fuels environmental injustice. In an attempt to dampen the effect, for example, the EU has adopted carbon emission allowances for several industries (e.g. energy) to favour the decarbonisation of the European economy (Lund 2007). In general, the policies originated and adopted target the productive sector and not directly the citizens affected by this phenomenon (e.g. unemployment). Consequently, general doubts (and doubts specific to justice concerns), continue to grow among scholars and policymakers about how countries can and/or should contribute to the common effort to reach global targets if they are calculated by production-based accounting, especially in the case of developing nations (Weber et al. 2008).

In addition to the justice limitations mentioned above, there are other fairness ‘challenges’ associated with the production-based accounting footprint. The principle of environmental accountability assumes that manufacturers have the scientific and/or technologic possibility to continually improve production processes. It also assumes that manufacturers can apply ‘greener’ production methods at a relevant scale while satisfying a growing need for products and services. This assumption disregards the factual challenges in technology transfer between nations and industries. As Avgerou and Walsham (2017) write, technology and knowledge transfers are particularly difficult when developing countries are the recipients. Furthermore, production-based footprint accounting presupposes increasing resource and/or energy efficiency. However, in many cases (e.g. steel), the technological limit is practically reached (Milford et al. 2013) proving such technologic optimism to be excessive.

To date, most environmental and socio-economic policies and potential measures (e.g. EU carbon emission allowances, ‘carbon tax’) have originated as a consequence of production-based accounting (e.g. production-based carbon footprint), which offers a matrix of justice considerations, especially regarding how to fairly prevent increased inequality deriving from ‘carbon’ offsetting measures (Böhringer et al. 2012). In sum, there are many justice implications and limitations that hinder production-based accounting in terms of being a just approach to determine environmental responsibility.

## The Responsibility Duality: Consumers as the New Environment Culprits

In a consumption-based footprint, the inventories include a value chain perspective, i.e. the system boundaries are open. Here, the data includes resource use, emissions and/or impacts caused by the production of goods and services consumed by the organisation or nation in question. This inclusion is independent of whether the resource use emissions and/or impacts occur inside or outside the organisational limits of the population or activity of interest. This footprint accounting method includes all the emissions, uses and/or impacts along the supply chains (Cazcarro et al. 2010; Larsen and Hertwich 2009). The justice consequence of this system boundary change is that consumers are ultimately responsible for any environmental impacts of the goods, services and energy imported from outside national borders and consumed in each country (Munksgaard and Pedersen 2001). As such, consumption-based accounting assumes the consumer is fully responsible for all the emissions, uses and impacts of the entire value chain.

Research has shown contrasting world trends in terms of carbon emissions: emissions embodied in trade have rapidly increased, whilst the gap between production emissions and the emissions associated with consumption have widened (Barrett et al. 2013). As Bastianoni et al. (2004: 255) warn: “without adequate incentives or policies, consumers are not likely to be sensitive […] to their environmental responsibilities, having, in fact, no consumption limits.” The fact that there is a positive correlation between consumption-based emissions and GDP (gross domestic product) (Lee and Lee 2009), makes this accounting method seem (more) just.

In comparison to production-based accounting, consumption-based accounting is more recent, so there are increased opportunities for methodological improvements (Afionis et al. 2017; Barrett et al. 2013). Nonetheless, the consumption-based principle has several flawed presuppositions. Firstly, it assumes that all consumers have access to environmental information about products and services. Secondly, it assumes that consumers understand such information, and thirdly, that they can actually choose the best alternatives. Making better choices requires the availability of ‘greener’ products, and populations must have the buying power to purchase the ‘greener’ alternatives. The third assumption disregards individual factors like personal indecision and incapacity to decide about trade-offs between resources, impacts and/or emissions. The prerequisites for shifting towards ‘greener’ consumption are particularly difficult to find in developing countries (due to e.g. price and availability constraints). Even in developed nations, there are cases where environmental education and environmental consciousness are not sufficiently developed in citizens to drive such transition (Franzen and Meyer 2010; Palmer et al. 1998), and in many cases, citizens from countries with high GDP do not know enough to make greener choices (see Tables 1 and 3). Consequently, it is reasonable to claim that it is unfair to centre the responsibility solely on the individual when there are significant socio-economic factors influencing the actions of consumers.

**Table 1:** Relevant moral limitations of production and consumption-based footprints

Characteristics	Production-based footprint	Consumption-based footprint
System frontiers and description	Incomplete: missing value chain associated emissions and global trade impacts	‘Demand-driven’ perspective of economy
	‘Supply-driven’ perspective of economy	
Justice agency	Personal responsibility for environmental impacts overlooked	Low or no accountability of companies and institutions
		Omission of relevant socio-economic factors that influence consumer behaviour
Developmental justice	Over-representation of manufacturing-intensive countries compared to post-manufacturing service economies (e.g. China vs. UK)	Undervaluing of political and institutional efforts to create a low impact economy (e.g. decarbonisation of industries)
	Support of institutional barriers to the achievement of international (carbon) protocols affecting predominantly developing countries (e.g. China, India, and Indonesia)	
Global justice	Exacerbation of North–South gap rhetoric: the Global North is seen in a positive light at the expense of the Global South (e.g. land use)	Assumption of an ‘ideal market’
		Potential misrepresentation of ‘non-trading’ economies due to lack of ‘greener’ consumption alternatives/substitutes (e.g. Cuba)

The consumption-based principle (and footprint)—of responsibility of the end consumer—relies on the general premise that the production of goods and services is (mainly or solely) driven by consumer demand. Such postulation is challengeable on the grounds of consumers ultimately not having the (full) capacity to be the ‘invisible’ hand, powerful enough to shape markets and turn them fairer and greener. If this were to be true, for instance, ocean oil spills would no longer exist due to the extensive environmental campaigns and public voices against this occurrence. Furthermore, even if improvements were to be made, consumption-based accounting would not become fairer as the basic accounting principle would not be altered: the end-consumer bears total responsibility. Table 1 describes briefly some inadequacies of production and consumption-based footprints focused justice issues.

## Sharing The Burden of Environmental Responsibility

As mentioned previously, both producer and consumer-based footprints have conceptual and justice limitations that hinder their results from being used in the wider contexts of environmental policymaking and environmental justice. However, despite described limitations, production and consumption calculation methods can still be relevant in pinpointing emitters and emissions fluxes. Using this information, new improved footprint accounting methods should be developed to target the question of environmental responsibility.

This article proposes an alternative approach to footprint calculation based essentially on the premise that footprints are policy-informing tools, attributing environmental responsibility to both companies and citizens. Responsibility is here understood as accountability for the adverse effects coming from free and rational choices. It is thus argued that both consumers and producers have an environmental responsibility since they can both negatively affect the environment through their choices (Fahlquist 2009). The responsibility is shared because the two actors contribute to single harmful outcomes (e.g. CO_2_ emissions, land, and water depletion). It is also shared because the contributions of each agent (consumers, producers) cannot be attributed to them based solely on causation. For example, the carbon emissions, land and water uses of the agricultural sector (producer) do not exist entirely due to individual (consumers) demand since this sector generates more food than is consumed. At the same time, carbon emissions and land and water use from consumption of certain food products (e.g. meat in Asia) are constrained by low production capacity. Furthermore, the responsibility of consumers and producers for their impact is distributed to them separately, rather than resting on them collectively, i.e. there is no meaningful eco-socio-economic collective entity that integrates both consumers and producers and, at the same, is responsible for the environmental impacts.

There are several ways of performing a shared production–consumption footprint (Rodrigues and Domingos 2008; Ferng 2003; Kanemoto et al. 2011). Due to the scope of this article, the different alternatives are not mapped exhaustively. As in the case of production-based or consumption-based accounting footprints, the majority of the methods for a shared production–consumption based footprint were developed for carbon. Nevertheless, there are no methodological impediments to use this approach for land or water footprints as well. Jiun–Jiun Ferng made one of the first attempts at designing a shared production-consumption footprint. In this case, the elements for calculating each agent’s share of the emissions are the consumption-benefit principle and the ecological deficit. The consumption- benefit principle states that the division of responsibility should be negotiated internationally while taking into account differences in national economic structures, consumption patterns and levels, and equal basic needs at a per capita basis. The ecological deficit quantifies the overuse of resources or the excess of emissions. The difference between human requirements and the carrying capacity is the footprint result (Ferng 2003). Another perspective for a shared production-consumption method was introduced by Pontus Cerin (2006) and Cerin and Karlson (2002). In these cases, the sharing parameter for each agent was calculated according to the degree of its influence over a value chain or benefit derived from any particular transaction. In 2007, Manfred Lenzen developed this approach further, but with a new focus on the economic opportunity of producers and consumers in engaging in economic transactions by means of division of responsibility (Lenzen 2007). In other words, responsibility for use, emissions and/or impacts was allocated differently, according to the added value of each element in the value chain. In any case, the methodologies presented so far do not have a strong theoretical justification i.e. the reasoning behind responsibility allocation is either arbitrary (Zaks et al. 2009; Lenzen 2007) or one-dimensional justifications traceable to economics (Cerin and Karlson 2002). Nonetheless, they demonstrate that there is the (mathematical) possibility of an improved accounting method that overcomes the limitations of the production and consumption-based footprints.

So far, there has not been a true discussion among the scientific community and the relevant stakeholders about the principles that should inform the division of responsibility. There is a strong possibility that under the prevalent economic perspective (Murphy et al. 1989; Rosenstein-Rodan 1943), shared responsibility approaches may not find new supporters, as confirmed by the lack of significant methodological advancements in recent years. Additional developments of this type of accounting method may well be more dependent on the evolution of economic, policy and justice concepts than on scientific progress.

## Justice elements for improving footprints

To generate an improved measure of environmental responsibility, it is necessary to look beyond the mathematical possibilities (shared production-consumption method) and search for the justice elements that can and should be incorporated. Since allocation responsibility implies, among other things, fair methods and results, developing a method based on a clear theoretical body of ethical work about justice seems a natural path. What is proposed here is the adoption of a shared production-consumption based accounting matrix, with coefficients of environmental responsibility of the agents based on justice reasons. So far, the stand is (sole) economic value dictating the share of environmental accountability (Lenzen et al. 2007; Kanemoto et al. 2011; Cerin 2006; Ferng 2003) which can be seen as a gross oversimplification. There are factors that determine the agent’s responsibility which go beyond economics and extend to the moral (justice) sphere (Fischer and Ravizza 1998; Forsyth 1992).

**Fig. 1:**
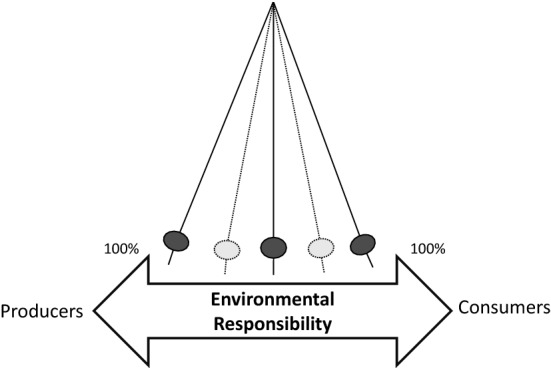
Representation of environmental responsibility attribution in the footprint method

The following proposed theoretical framework is an opportunity to start a consistent and grounded discussion about the premises which should be behind a just environmental responsibility allocation. The framework suggested here has the novelty of integrating the specificities of the agents in their national contexts, i.e. the ‘just allocation’ of responsibility should be calculated according to specific characteristics of producers and consumers of each nation. Figure 1 represents how, in footprints, environmental responsibility can be theoretically divided among the agents. To overcome the limitations of the accounting methods, ‘just’ footprint includes (all) the agents responsible for environmental impacts and resource use while contextualising the capacity of producers’ and consumers’ to consider and/or change to better environmental alternatives. Table 2 describes the characteristics of ‘just’ footprint in response to the justice limitations of production and consumption-based accounting footprints.

**Table 2:** Justice characteristics of production-consumption based ‘just’ footprint

Justice limitations of footprint accounting	Production-consumption based ‘Just’ footprint
System frontiers and description	Inclusion of emissions, impacts and resource use embodied in trade
	Recognition of mutual influence of production and consumption in global trade
Justice agency	Contextualized shared responsibility for environmental impacts, emissions and resource use
	Integration of relevant socio-economic factors that influence producer action and consumer behaviour
Developmental justice	Integration of eco-socio-economic factors
Distributive justice (nations and individuals)	Incorporation of indicators of a nation’s wealth and individual income disparity
Global justice	Increased neutrality towards different socio-economic models

Table 3 enunciates the moral justification for the inclusion of the parameters integrated in the ‘just’ footprint calculation framework. These parameters derive from the concept of environmental justice described in the introduction and focus on the factors that (can) directly affect the agents involved (producers and consumers). It is important to make clear that ‘producers’ and ‘consumers’ are defined and understood here according to the tradition of input–output analysis (Miller and Blair 2009). The suggested parameters do not exhaust the justice implications for individuals and groups seen under other traditions, such as climate and environmental justice. For example, they do not tackle the disproportionate burden of climate change on racially-diverse communities. The parameters included in the ‘just’ footprint calculation are ‘adjusted’ to the characterisation of input–output national accounting of these categories. In input–output analysis, the industries and service sectors employing economic activities are considered ‘producers’ while ‘consumers’ aggregate households and government levels. Note that for the purposes of production, producers utilise goods and services from other producers and therefore can also be regarded as (intermediate) consumers in the input–output model (Miller and Blair 2009). The nature of such definitions greatly narrows the environmental justice implications that can be associated with these categories since they need to be quantifiable and the data need to be available in the input–output national accounting system.

**Table 3:** Justification for inclusion of parameters in ‘just’ footprint calculation

Agent	Parameter	Justice justification
Producers	Technological improvement capacity	Institutional obligation of (re)-design towards improved (environmental) justice standards (Rawls 1971)
	Technological sectorial improvement capacity	The economic possibility of production sectors to use the best available ‘greener’ technology (Van Marrewijk 2003; Dahlsrud 2008)
	Availability of ‘greener’ substitute goods for production	The existence of alternatives is pre-requisite for (re)-design towards improved (environmental) justice standards (Rawls 1971; Cohen 1989)
Consumers	Environmental awareness	The individual *sense of justice* is the base of consistent decisions in the quest of what is just (Rawls 1971). Education has the mandate to strengthen justice and environmental awareness (and action) (Apple 2009)
	Purchasing power	Monetary resources are pre-requisite for acquiring products. Low/deficient economic resources diminish the freedom to act accordingly to justice principles (Rawls 1971; Glickman 2009)
	Availability of ‘greener’ substitute goods for consumption	The existence of alternatives is a pre-requisite for free, reasonable and rational choices (Rawls 1971)

In practical terms, in the proposed ‘just’ footprint, the environmental responsibility quota of each agent is defined according to the theoretical and practical possibility of producers and consumers to diminish their impacts, emissions or resource use, framed by the eco-socio-economic conditions of each nation or territory. It is proposed that the producers’ environmental responsibility, calculated via a ‘just’ footprint, should be a function of the (1) technological improvement capacity, (2) technological sectorial improvement capacity and (3) availability of ‘greener’ substitute goods and services. In the case of consumers’ environmental responsibility, it should be calculated as a function of the (1) general environmental awareness of the population, (2) their purchase power (corrected by the inequality level) and (3) availability of ‘greener’ substitute goods and services.

In other words, the ‘just’ footprint combines measures which go beyond the systems of national accounts parameters and includes others that ensure a fair characterisation of the system. By doing this, the greatest strength is to account not only for what the agents are using, impacting and emitting (‘classical’ footprint) but also what they are capable of, and willing to improve in their environmental performance, in real life situations, which is the national context where they operate. It seems unreasonable to directly blame the consumers of an impoverished and/or underdeveloped nation for environmental impacts if they can only afford to buy the most readily available and cheapest items, which might be originated from ‘dirty’ production methods. The same reasoning holds for companies that cannot access the best technology of production because they operate in a country tarnished by war or under international sanctions. Table 4 shows the parameters used in the ‘just’ footprint for calculating carbon emissions, water and land use. Potential indicators or indexes that can operationalise the parameters are suggested for each one of the parameters. Some of the indicators can be associated with developmental (e.g. gross fixed capital formation) and environmental justice (environmental awareness index) and environmental vulnerability (water exploitation index). Since the ‘just’ footprints are to be calculated per nation, the proposed indicators and indexes pertain to accounts available to countries.

**Table 4:** Parameters, indicators and indexes included in ‘just’ footprint calculation

Agent	Footprint parameter	Resource/environmental impact		
		Carbon	Water	Land
Producers	Technological improvement capacity	^1^Lowest carbon intensity production chain	^1^Lowest water use and aquatic pollution production chain (incl. blue* and grey water**)	^1^Lowest land use
	Indicator(s)/index(es)	GHG emissions	^2^Water exploitation index^a^	Artificial land or built-up area^c^
			^2^Water quality index^b^	
	Technological sectorial improvement capacity	Economic sectorial capacity to invest in technological improvement		
	Indicator(s)/index(es)	Gross fixed capital formation (as percentage of GDP)^c^		
	Availability of ‘greener’ substitute goods and services	Technology transfer capacity		
		Import/export restrictions		
	Indicator(s)/index(es)	Import partner share^e^		
		FDI and technology transfer^f^		
Consumers	Environmental awareness	Degree of recognition of global warming	Degree of recognition of water scarcity and pollution	Degree of recognition of land misuse
	Indicator(s)/index(es)	^3^Education index^g^		
		^3^Environmental awareness index^h^		
	Purchasing power	Economic capacity of individuals and/or households to buy ‘greener’ goods and services		
	Indicator(s)/index(es)	Purchase power parity (GDP_PPP_/cap)^i^		
	Availability of ‘greener’ substitute goods and services	Degree of economic openness		
	Indicator(s)/index(es)	Openness to trade ^j^		

*Blue water is the ‘surface water and groundwater required (evaporated or used directly) to make a product’ (Grace Communications Foundation 2016)

**Grey water is ‘the amount of freshwater required to mix and dilute pollutants enough to maintain water quality according to governmental standards (e.g. US Clean Water Act) as a result of making a product’ (Grace Communications Foundation 2016)

^1^Benchmarked against the best (current) production example within each sector, i.e. the country whose production sector has the lowest impact/resource use is used as the example of improvement potential for the rest of the countries

^2^An established water pollution index was not found in the literature

^3^Established indicators or indexes concerning the social awareness of global warming, water scarcity or land misuse were not found in literature

^a^(Lallana and Marcuello 2004); ^b^(Harkins 1974); ^c^(Giljum et al. 2013); ^d^(The World Bank 2018c); ^e^(WITS 2018a); ^i^(The World Bank 2018a); ^g^(UNDP 2018); ^h^(Kokkinen 2014); ^i^(The World Bank 2018b); ^j^(WITS 2018b)

## Method

This section describes how a ‘just’ footprint can be calculated. Although a full implementation of the concept is outside the scope of this article, a simple example is presented to illustrate a potential implementation.

Suppose a two-region economy. Each of the regions has the same number of economic sectors and the regions trade goods and services with each other. The production-based footprint *F* of either of the regions is given by the sum of emissions associated with production for domestic consumption, emissions associated with production for export, and emissions by final demand sectors such as government and households:

1 $$F_{n}^{prod} = F_{n}^{dom} + F_{n}^{exp} + F_{n}^{fd}$$

From a consumption perspective, emissions are accounted for via summing domestic emissions, emissions embodied in the imports, and emissions from final demand sectors.

2 $$F_{n}^{cons} = F_{n}^{dom} + F_{n}^{imp} + F_{n}^{fd}$$

Note that in a two-region economy, the export from region 1–2 equals the import from region 2–1 and vice versa. This implies that the consumption-based footprint for region 1 can be re-written as follows:

3 $$F_{1}^{cons} = F_{1}^{dom} + F_{2}^{exp} + F_{1}^{fd}$$

As emissions associated with production for both domestic consumption and emission associated with final demand remain equal, it is clear that the difference between consumption and production perspective lies in the treatment of emissions embodied in the trade flows between the two regions. Therefore, a ‘just footprint’ should aim for a re-allocation of these embodied emissions to each of the regions, to represent a shared production-consumption perspective. One such allocation could be the following, where part of the emissions embodied in exports and part of the emissions embodied in imports are allocated to both regions.

4a $$F_{1}^{just} = F_{1}^{dom} + \alpha F_{2}^{exp} + \left( {1 - \beta } \right)F_{1}^{exp} + F_{1}^{fd}$$

4b $$F_{2}^{just} = F_{2}^{dom} + \beta F_{1}^{exp} + \left( {1 - \alpha } \right)F_{2}^{exp} + F_{2}^{fd}$$

The crux to a just accounting framework lies in a proper establishment of the weights *α* and *β* presented in Eq. 4. Note that these weights can be established from both a producer perspective (i.e. through exports) and consumer perspective (i.e. through imports) and both perspectives should be included in the calculation of weights *α* and *β.*

In Table 4, several indicators were presented that reflect various aspects of consumer or production accountability. In more general terms, one could think of *i* consumption perspective indicators C, and *j* production perspective indicators P, for respectively region 1 and region 2. *α* and *β* can subsequently be defined as follows:

5a–5d $$\begin{aligned} \alpha = \frac{{\mathop \sum \nolimits_{i} C_{1}^{i} }}{{\mathop \sum \nolimits_{i} C_{1}^{i} + \mathop \sum \nolimits_{j} P_{2}^{j} }} ;\quad \left( {1 - \alpha } \right) = \frac{{\mathop \sum \nolimits_{j} P_{2}^{j} }}{{\mathop \sum \nolimits_{i} C_{1}^{i} + \mathop \sum \nolimits_{j} P_{2}^{j} }} \hfill \\ \beta = \frac{{\mathop \sum \nolimits_{i} C_{2}^{i} }}{{\mathop \sum \nolimits_{i} C_{2}^{i} + \mathop \sum \nolimits_{j} P_{1}^{j} }};\quad \left( {1 - \beta } \right) = \frac{{\mathop \sum \nolimits_{j} P_{1}^{j} }}{{\mathop \sum \nolimits_{i} C_{2}^{i} + \mathop \sum \nolimits_{j} P_{1}^{j} }} \hfill \\ \end{aligned}$$

Note that a normalization of the indicators might be required to ensure that all indicators have the same relative weight in the calculation of *α* and *β.*

Next, several scenarios pertaining to regions 1 and 2 and the outcome of the just footprint calculation under *ceteris paribus* conditions are discussed to demonstrate the behaviour of the accounting model.

- *Scenario 1:* Region 1 implements cleaner technologies and therefore reduces the indicator for greenhouse gas emissions intensity (reflecting a change in producers’ parameters- see Table 3).
  - A decrease in GHG emissions intensity will lead to an increase in *β*. As a result, fewer of the emissions associated with exports will be allocated to region 1. This effect, in combination with the decrease in domestic emissions, leads to a decreased ‘just’ footprint.
- *Scenario 2:* Region 1 has more capital available for investment, represented by gross fixed capital formation as a percentage of GDP, and could, therefore, invest in technologies to produce with lower environmental impacts (reflecting a change in producers’ parameters- see Table 3).
  - An increase in this indicator will decrease *β* and as a result, the ‘just’ footprint of region 1 will increase as more of the emissions embodied in exports are allocated to the region.
- *Scenario 3:* Region 2 opens up to trade and increases the affluence of their citizens as reflected by increased purchasing power parity (reflecting a change in consumers’ parameters- see Table 3).
  - An increase in these two indicators will lead to an increase in *β* resulting in an increase in the ‘just’ footprint of region 2 as more of the emissions embodied in imports are allocated to the region.

The above-illustrated scenarios exemplify that the calculation of weights behaves correctly in re-allocating emissions for the ‘just’ footprint. It is important to note that the above-described model allows for the inclusion of more indicators than the ones described in Table 4. Establishing a final set of indicators is not the purpose of this article since the aim here is to show a generic theoretical framework. The choice of the indicators can and should be done by relevant stakeholders, such as the United Nations, affected communities or countries, in an inclusive and democratic process.

Since the proposed model is based on MRIO tables, like ‘classical’ footprints, it has the same general weaknesses and strengths (Galli et al. 2012). However, the integration of justice parameters (*α* and *β*) in the calculation of the ‘just’ footprints strengthens the acknowledgement by scientists, economists and engineers that eco-socio-economic systems are regulated and operate in a larger scale, which cannot be reduced to economic parameters (e.g. environmental awareness). Such recognition creates a stronger basis for the acceptance of the footprint results. As mentioned before, the suggested parameters are not intended to express all relevant justice issues associated with environmental responsibility but rather demonstrate that is possible to account for at least some justice elements.

The suggested accounting method has straightforward and intuitive premises, which are easily identifiable by public opinion. In turn, this makes the results easier to integrate into policymaking; by articulating economic and environmental data with ethical premises, this model bridges distinct knowledge areas and values that are paramount to sustainable development as, for example, defined by the United Nations (Vasconcellos Oliveira 2018). A sustainable society demands integrated solutions from scientists, economists and engineers, and this model is a small contribution to this holistic perspective.

## Discussion and Conclusions

This article argues that environmental indicators, especially footprints, influence the way human impact on the environment is perceived. Footprints shape the opinions and actions of environmental scientists, policymakers, media and the general public. Despite this influence, scientists and engineers still struggle to deliver a desired integrative accounting system.

Environmental policies and environmental justice debates are not immune to the influence of footprints, especially when strategies need to be put in place to mitigate environmental impacts. Two cornerstones of effective policies are (1) knowing who originates the environmental problems and (2) to what extent they can shift towards lower impacts. Despite being easily understandable, and therefore communicated to and by policymakers, footprints so far do not live up to the previously mentioned expectations.

The footprint’s dichotomic perspective on each agent’s environmental responsibility is insufficient and potentially harmful for policy purposes. The producer and consumer-based methods footprints give an insufficiently accurate picture of reality, distorting the environmental justice debate. Inequality in water use or (inter)national accountability for sharing burdens of carbon emissions are examples of environmental justice topics that need a nuanced description of the phenomena. As Steininger et al. (2014) write, neither consumption-based nor production-based policies have improved climate change. Nevertheless, despite the limitations of consumption-based and production-based footprints, it is not likely or desirable to ignore information originated by footprints, especially when it concerns the variation and destination of environmental fluxes (e.g. pollutants) and resource use, and of environmental impacts.

In the last years, there has been a methodological stagnation in footprint calculation. Still, there is a real possibility to improve the weak points of footprints and re-configure them correctly and efficiently to support environmental policies and also to contribute theoretically and practically to environmental justice. Regardless, it is necessary that the footprint method be a sound one. The policy legitimacy to use footprint results is dependent on footprints that are scientifically accurate and just. Consequently, there is a moral imperative to develop methods that guarantee these attributes.

The proposed ‘just’ footprint is a methodological framework that attempts to conjugate the most ‘just’ scientific accounting process (shared producer–consumer method) with elements that concern agency, developmental, distributive and global justice. It is important to make clear these elements do not try to cover all relevant dimensions and issues concerning each type of justice. For example, power distribution among agents, or the effect of carbon emissions or misuse of water or land in worst-off groups are relevant matters for distributive and global justice that are not covered here. Due to the nature of the data available for this footprint and also the way it is calculated, only a few parameters were incorporated. The inclusion of justice parameters in the proposed footprint framework focuses on and contextualises the responsibility of both producers and consumers for their actions while setting in stone a detailed perspective of environmental agency and responsibility.

In general terms, the ‘just’ footprint is ‘sensitive’ to opportunities for environmental improvement. When companies and institutions of a particular region have power (economic capacity) and the means (technology) to produce ‘greener’ they are more accountable for their impacts. The same reasoning holds for individuals who have and know about ‘greener’ alternatives and have the economic capacity to buy them. Distinct from other accounting methods, the described ‘just’ footprint discloses its justice assumptions. Consequently, the agents (scientists, engineers, politicians) who choose to use this method become responsible for pushing forward an environmental accountability perspective based on individual and institutional capacity for (green) shift. Additionally, they turn into conscious agents of an environmental narrative centred on justice for people and the environment. The ‘just’ footprint is an example of the necessary integration of scientific disciplines to overcome the multifaceted challenges of sustainability. Furthermore, it reinforces a trend in science and engineering of creating knowledge and implementing solutions that meet societal needs (justice) and accommodate moral differences. By drawing on manifest justice premises (variables), the proposed footprint can be adjusted to the ethical evolution of the justice theories themselves. This translates into a lessening of bias and an increase in science transparency.

The (re-)design of a well-accepted environmental assessment tool to meet minimal justice standards thus creates a unique chance of reinforcing policymaking based on scientific and moral foundations. The ‘just’ footprint shows which variables policymakers can influence for positive environmental and justice improvement. When countries and regions stimulate investment in ‘green’ technology and facilitate access to it, producers (e.g. companies) have the opportunity to decrease the footprint of the region by installing ‘greener’ technologies. When countries and regions increase citizens’ accessibility (economic and material) to ‘greener’ products and services and invest in environmental education and awareness, consumers assume a higher responsibility for their impacts.

The ‘just’ footprint also contributes to a successful environmental strategy in several ways: it facilitates and expedites the use of scientific information, it sanctions the agents involved in the process, and above all, it legitimises the political process and its outcomes. Policymaking procedures, especially the democratic ones, frequently suffer from validity and authority shortcomings undermining their efficiency (Papadopoulos 2013). The situation is particularly acute in environmental issues since the high degree of complexity and numerous trade-offs create extra barriers to a successful implementation of strategies. It is imperative to reconnect the objects of policymaking (citizens, institutions, nations) to the agents who produce legislation. Justice is a universal value that can help in this task. The creation of more ‘just’ policymaking tools increase the chances for generalised acceptance of measures, even if they might require additional effort from societal agents.
